# Artificial intelligence as a tool to enhance social interventions in reducing crime

**DOI:** 10.3389/frai.2025.1661266

**Published:** 2025-09-25

**Authors:** Haya H. Tarawneh, Niveen Z. Halalsheh, Bahjat Abu Sulaiman, Huda A. Alhajjaj, Nesreen N. Atieh

**Affiliations:** 1 The University of Jordan, Aljubeiha, Jordan; 2 Department of Social Work, Faculty of Arts, The University of Jordan, Amman, Jordan; 3 Department of Journalism, Media and Digital Communication, School of Arts, The University of Jordan, Amman, Jordan; 4 Educational and Psychological Counseling, Saudi Arabia, Saudi Arabia

**Keywords:** artificial intelligence, social interventions, crime, enhance social, tool

## Abstract

**Introduction:**

This study explores the significant role of artificial intelligence (AI) in crime reduction, identifies the main challenges hindering its implementation, and examines differences in coping strategies between individuals in Jordan and Saudi Arabia.

**Methods:**

The research surveyed 170 AI professionals, equally divided between the two countries, with an average age of 45.2 years. Data were collected using a specially designed questionnaire assessing perceptions of AI, barriers to adoption, and coping mechanisms.

**Results:**

The findings indicated that AI plays a significant role in crime reduction. High levels of challenges were reported in implementing AI, and coping strategies related to AI in crime reduction were also assessed at a high level. No statistically significant differences were found in the level of challenges facing AI between Jordanian and Saudi participants.

**Discussion:**

This research contributes to understanding AI’s practical applications in crime prevention and provides valuable insights for policymakers to strengthen AI adoption and overcome existing barriers in the region.

## Introduction

Crime has become increasingly complex in the modern era, with rapid advancements in communication, technology, and social structures contributing to the emergence of new forms and methods of criminal activity. Traditional law enforcement techniques, while essential, often fall short in addressing these evolving challenges. These limitations have highlighted the need for innovative solutions capable of responding to crime more proactively and efficiently.

In recent decades, a significant technological revolution has taken place across many scientific and practical fields, including crime prevention, criminal investigations, evidence collection, and overall public safety. Among these advancements, Artificial Intelligence (AI) has emerged as a powerful tool that can fill critical gaps in traditional approaches and significantly improve the effectiveness of crime prevention strategies.

AI technologies have greatly enhanced investigators’ ability to track crimes quickly and accurately. Beyond that, AI is used to gather evidence, draw conclusions about crimes and their perpetrators, and help unravel the complexities behind criminal activities (Bharti et al., 2024, p. 873). These technologies provide reliable and precise information that aids in reducing and preventing crime. Some of the most notable tools include facial recognition systems, audio recording apps, remote listening devices, and suspect identification methods. These innovations help law enforcement uncover more crimes and potential security threats, ultimately boosting the safety and security of communities (Mieleszczenko-Kowszewicz et al., 2023, p. 108).

Although crime is a natural part of any society, its escalation beyond acceptable limits poses a serious concern. The increasing diversity and sophistication of criminal methods—shaped by social, economic, political, technological, and communication-related changes—call for a reassessment of current strategies and the development of more effective solutions. AI plays an important role in this context, as it enhances human capabilities and enables smart systems to detect danger zones and respond quickly and efficiently. This leads to better control, higher efficiency, and faster identification of criminals (Al-Matroushi, 2024, p. 2).

According to Sinha (2025), AI plays a crucial role in spotting, identifying, and predicting crimes, making it easier to control and prevent them. AI has become a cornerstone for security and police agencies, helping analyze crime trends, patterns, and prevention strategies (Al-Babli, 2019, p. 60).

Crime itself is a broad concept, encompassing many different types. Property crimes include burglary, theft, and shoplifting, while violent crimes cover murder, kidnapping, sexual assault, and more. Crime rates can vary widely depending on the kind, severity, and impact of the offense (Vinuesa and Sirmacek, 2025, p. 926).

Recent studies indicate that digital transformation has become a critical driver for innovation, efficiency, and the overall improvement of services across multiple sectors (Al-Aroud et al., 2025). The Ministry of Digital Economy and Entrepreneurship (2023) emphasizes that well-structured policies supporting digital transformation are essential for building robust digital infrastructure and fostering a culture of entrepreneurship. In addition, Musa (2023) notes that investment in advanced digital technologies not only enhances operational efficiency but also significantly improves the user experience and service delivery. Furthermore, Alkhattabi (2025) highlights that integrating comprehensive digital education and training strategies is crucial for ensuring that these transformations are sustainable and effectively embedded within organizational practices over the long term. Collectively, these findings demonstrate that successful digital transformation requires a combination of policy support, technological investment, and workforce readiness.

## Purpose and objectives of the study

In light of the tremendous revolution in the field of technology and modern technology, and the significant increase in the uses of artificial intelligence in all areas of life, and its prominent role in societies in all fields such as security, work, economy, communications, wars, reducing crime and other various specializations through which human work can be facilitated and accomplished with accuracy and efficiency that exceeds human efficiency and ability (Chaturvedi et al., 2024, p. 195), the purpose and objectives of the current study are to identify the role of artificial intelligence and its importance in reducing crime, the challenges it faces, and the styles of coping with artificial intelligence.

In addition, this study aims to conduct a comparative analysis of the perceived roles, challenges, and coping mechanisms related to AI adoption in law enforcement between individuals in Jordanian and Saudi societies, two nations undergoing rapid digital transformation.

This comparative focus seeks to highlight similarities and differences in experiences and responses, thereby contributing to a deeper understanding that may inform tailored strategies for each context.

## Significance of the study

The importance of the current study, both theoretically and practically, is evident in the appropriate selection of study variables, which have not been previously studied in the Jordanian context, to the best of the researcher’s knowledge. It is also a serious attempt to understand artificial intelligence and its importance in enhancing social interventions to reduce crime, identify the challenges facing AI applications in reducing crime, and compare the methods used by individuals in Jordanian and Saudi societies to overcome such challenges. This will contribute to the development of treatment and guidance programs and plans to mitigate crime using AI, and open up horizons for researchers for future studies that will enable them to understand the role and impact of AI in various areas of life.

## Study terms

- Artificial Intelligence (AI): is an artificial entity that solves complex problems. Such a system is generally assumed to be a computer or a machine through which computer science and physiology can be integrated with computer science to be able to achieve the world’s goals and the ability to create imaginative thinking in memorization and understanding, recognize patterns, make choices to adapt to change and learn from experience, and make computers behave like humans and in much less time than it takes a human (Borana, 2024, p. 64). It is also defined by Boden (2024, p. 27) as “a computer-programmed machine that uses specific algorithms and procedures to perform a particular task or action. This programmed machine gets automatic inputs and applies the same according to the program.”
- Social Interventions: are the social conditions surrounding the individual and affecting his recidivism, such as family circumstances and the surrounding environment, society’s view of those released, i.e., the extent to which society accepts or rejects him, the group of friends, the social environment, and other conditions that may play a role in returning to crime (Wang et al., 2023, p. 899). Crear-Perry et al. (2022, p. 230) defined it as all the forces, influences, and specific conditions prevailing in society related to the organization of society, and these forces and influences interact with each other, leading to results that appear in the behavior of the individuals of the society in which they exist.
- Crime is a social phenomenon that is an anti-social behavior, a form of deviation from normal behavior and a departure from the standards, values, traditions, and norms of society (Warnock-Parkes et al., 2024, p. 835).

## Artificial intelligence and its role in monitoring, detecting and predicting crimes

Automated models of AI techniques can be used to predict or prevent future crimes before they occur, thereby ensuring public safety. Unlike traditional methods that often rely on static historical data or manual hotspot mapping, AI employs machine learning algorithms to analyze complex, multi-dimensional datasets—including time, location, type of crime, and social factors—allowing for more dynamic and precise identification of crime hotspots and patterns. This enables police departments to optimize resource allocation based on predictive insights (Shermila et al., 2024, p. 108).

AI techniques also facilitate quantitative analysis of large datasets collected by public safety departments to extract meaningful patterns and forecast future events. For example, Kim et al. (2023, p. 415) analyzed crime data from Vancouver over 15 years using heat maps combined with various AI methods to predict areas prone to criminal activity.

Smart technologies analyze specific inputs through advanced algorithms that provide highly accurate and actionable results, helping law enforcement agencies prepare for and prevent crimes proactively rather than reactively (Odaybat and Abu Ayyadah, 2023, p. 212).

Regarding AI’s role in crime monitoring, several studies have confirmed its effectiveness. Rouhollahi (2022) found AI to be a powerful tool in detecting crimes broadly and financial crimes specifically, such as money laundering. Ritesh and Kumar (2022) demonstrated AI’s contribution to fingerprint detection and facial recognition, crucial for crime investigation. Powelson (2022) highlighted AI’s significant role in enhancing the effectiveness of crime-fighting programs. Udaybat and Ayyadah (2023) emphasized AI’s importance in crime monitoring and prevention. Al-Marghilani (2024) showed AI’s role in suspect identification and precise crime scene mapping, while Al-Shahri (2024) pointed to AI’s critical contributions in crime detection, perpetrator identification, evidence gathering, and overall societal security enhancement.

By continuously learning from new data, AI models adapt to emerging crime trends, making them more effective than traditional static methods in addressing the evolving nature of criminal behavior.

### Artificial intelligence and its role in preventing and reducing crimes

Akerkar (2025, p. 975) explained that artificial intelligence technologies assist the criminal justice system by identifying the true perpetrator of an incident. Through their complex programming and the use of specific algorithms, they can uncover the mystery surrounding any incident, using the data they obtain. By photographing the crime scene and studying the health status of the accused, they can prove whether or not they are capable of committing the behavior constituting the crime, with greater accuracy than humans. Through facial recognition technologies and cross-checking this with the country’s camera databases, they can prove, in a moment, that the accused was in a location other than the crime scene, thus acquitting them of the charge against them. Artificial intelligence technologies have contributed to providing important and accurate information that helps reduce and prevent crimes. Among the most important of these technologies are facial recognition, audio recording applications, remote listening, and identifying perpetrators, which helps uncover more crimes and security threats. The implementation of the smart crime prediction system, the “Smart Crime Scene Program,” also enhances the safety and security of citizens (Al-Shahri, 2024, p. 74).

Judicial officers resort to using artificial intelligence technologies by downloading and analyzing surveillance cameras, examining all locations, shops, or streets surrounding the crime scene, and identifying the movements of all individuals at or near the crime scene. This is done with the aim of identifying suspects and subjecting them to investigation.

There are programs that can analyze all this information and images accurately and in a very short time, objectively and without personal bias (Davenport and Kalakota, 2024, p. 95). Among the technologies used by judicial officers in investigations and evidence gathering are smartphone data analysis, monitoring calls made at the time of the crime to a large number of cellular networks, and determining the location from which the suspect made the call. This is facilitated by activity tracking technology on smart devices, which facilitates investigations, gathers evidence, and ensures the accuracy of the evidence collected. This contributes to achieving justice.

Without artificial intelligence technologies and software in such cases, a human cadre consisting of hundreds of people would not be able to analyze the signals sent from phones, analyze voices, and monitor the calls. Artificial intelligence technologies have been credited for uncovering the threads of the most difficult and dangerous crimes, identifying the perpetrators, and bringing them to justice (Ryan, 2024, p. 2).

Numerous studies have addressed the variable of artificial intelligence and its role in preventing and reducing crimes, such as the study (Selma, 2025), which aimed to identify the role of artificial intelligence technologies in detecting crimes and security threats. The study results showed that artificial intelligence plays a significant role in detecting and preventing crimes and security threats, and that the use of artificial intelligence technologies paves the way for combating, detecting, and preventing cybercrimes. The study (Remian, 2025) aimed to identify the role of artificial intelligence in predicting crime. The study results showed that artificial intelligence plays a significant role in predicting the likelihood of future crimes through archival data and statistical methods, which contributes to reducing crimes and increasing public safety.

### Artificial intelligence in the Hashemite Kingdom of Jordan: between reality and challenges

Recognizing the tremendous opportunities offered by the Fourth Industrial Revolution and its significant role in improving human life, the Hashemite Kingdom of Jordan continues its pursuit of sustainable economic development by strengthening and embracing societal strengths based on adherence to values, building on achievements, and leveraging available opportunities to build a modern civil society characterized by justice, equality, equal opportunities, and respect for human rights. This is achieved through the optimal use of knowledge and technological tools. This is in addition to avoiding concerns that may limit the adoption of AI-based technological solutions, such as weak accountability, unclear decision-making mechanisms, or unfair and biased data analysis. In implementation of the provisions of the Jordanian Artificial Intelligence Policy 2020, the Ministry of Digital Economy and Entrepreneurship has prepared a national charter for AI ethics in cooperation with relevant authorities from the public, private, and academic sectors, civil society institutions, and security agencies (Ministry of Digital Economy and Entrepreneurship, 2025, p. 7).

The Jordanian Artificial Intelligence Strategy and Implementation Plan 2023–2027 is an extension of previous strategies and policies regulating digital transformation and digital technology, implemented by the government. It aligns with global trends in adopting artificial intelligence, with the goal of accelerating social and economic development for citizens in the most important areas, such as health, education, social protection, employment, and welfare. This is achieved by creating a supportive and stimulating environment for the development of artificial intelligence systems at the national level, in addition to building capacity, developing human skills, and raising the efficiency of education at all educational levels (Ministry of Digital Economy and Entrepreneurship, 2025, p. 5).

The Hashemite Kingdom of Jordan established the Cybercrime Unit in Jordan under the umbrella of the Public Security Directorate in 2015. This was to address the challenges posed by the rapid development of information and communications technology and the continuous increase in the use of the internet and digital technology in daily life and business. This unit aims to combat cybercrime and protect Jordanian society from threats by analyzing and assessing technology-related threats and crimes, implementing the necessary preventive and investigative measures to address these crimes, and prosecuting suspects (Al-Rawashdeh, 2025, p. 123).

Regarding the challenges facing the application of artificial intelligence in the Hashemite Kingdom of Jordan, (Muqaddadi, 2024, p. 333; Al-Aroud et al., 2025, p. 165) explained that the most prominent of these challenges are material and technical challenges, followed by a lack of awareness of the importance of using these technologies, and the scarcity of training courses that motivate workers to employ artificial intelligence technologies.

### Artificial intelligence in the Kingdom of Saudi Arabia: between reality and challenges

The Kingdom of Saudi Arabia has focused on investing in this advanced technology through its Vision 2030, which seeks continuous development and global competitiveness based on a wealth of innovation and creativity. Therefore, the Kingdom has sought to launch a unique strategy that has helped improve institutional and governmental performance, accelerate achievements, and create a pioneering work environment characterized by innovation and creativity. The government has not limited itself to this, but has established the Saudi Data and Artificial Intelligence Authority and launched the National Strategy for Data and Artificial Intelligence (Al-Shahri, 2024, p. 74).

The Kingdom of Saudi Arabia has anticipated the importance of artificial intelligence and worked to prepare for it early, entering the world of digitization and communications, developing digital infrastructure, and enhancing reliance on data and artificial intelligence as catalysts for rapid development. The Kingdom has approved nearly $20 billion in investment in this field until 2030, as part of an ambitious plan aimed at diversifying the economy and reducing dependence on oil (Salama and Hinton, 2023, p. 41).

The Kingdom of Saudi Arabia has worked to utilize artificial intelligence technologies in all aspects of practical, economic, cultural, and social life, including public security and police work to enhance the security and public safety of individuals within the Kingdom, and to control security violations. It has also worked in research and development through the establishment of the Saudi Data and Artificial Intelligence Authority (SDAIA), which specializes in implementing research projects and promoting innovation. It has also worked in health, establishing the Artificial Intelligence Center in the Kingdom, and in crime prevention, detection, and prediction, identifying criminals, activating the electronic operating system for facial recognition at immigration offices, and collecting evidence about crimes and perpetrators (Saudi Ministry of Interior, 2024; Saudi Data and Artificial Intelligence Authority, 2024; Saudi Research and Information Center, 2021; Al-Babli, 2019; Al-Ubaidi, 2024).

There are many challenges facing the application of artificial intelligence in the Kingdom of Saudi Arabia, including a lack of awareness of the importance of using artificial intelligence in reducing crimes, the prevailing belief among some individuals that the role of artificial intelligence may lead to the overriding role of the human element, weak awareness among individuals of the importance of employing artificial intelligence in all fields, resistance by some individuals to the idea of introducing artificial intelligence in combating crimes and their lack of conviction of its importance, a lack of training programs to qualify individuals to use artificial intelligence technologies, a lack of financial allocations for employing artificial intelligence in all fields, the failure to link artificial intelligence to education and its failure to generalize it at all levels, the lack of availability of specialists and experts in artificial intelligence technologies, and the lack of an adequate infrastructure for information and communication technology (Al-Saud, 2020; Al-Bashar, 2022; Al-Abbasi and Al-Ghamdi, 2024; Turki, 2024; Alkhattabi, 2025).

## Hypotheses

Upon reviewing the theoretical foundations and prior research related to the current study’s variables—artificial intelligence and crime—the researchers found that these topics have attracted considerable attention across various academic and professional disciplines. Scholars have explored them from diverse perspectives, using a range of approaches and objectives. This review also enabled the researcher to formulate the following hypotheses:

1. Artificial intelligence plays a significant role in reducing crime.
2. There are notable challenges that hinder the effectiveness of artificial intelligence in combating crime.
3. There are Styles Coping Artificial intelligence in reducing crime.
4. There are differences between Jordanian and Saudi societies in how they address the challenges associated with artificial intelligence.

## Methodology of the study

This is a descriptive comparative approach, where the researcher seeks, through her current study, to describe and analyze artificial intelligence and identify its role in enhancing social interventions to reduce crime, as well as to identify the challenges facing artificial intelligence applications in reducing crime, as well as to compare the methods used by individuals in Jordanian and Saudi society to overcome such challenges.

**Figure 1:**
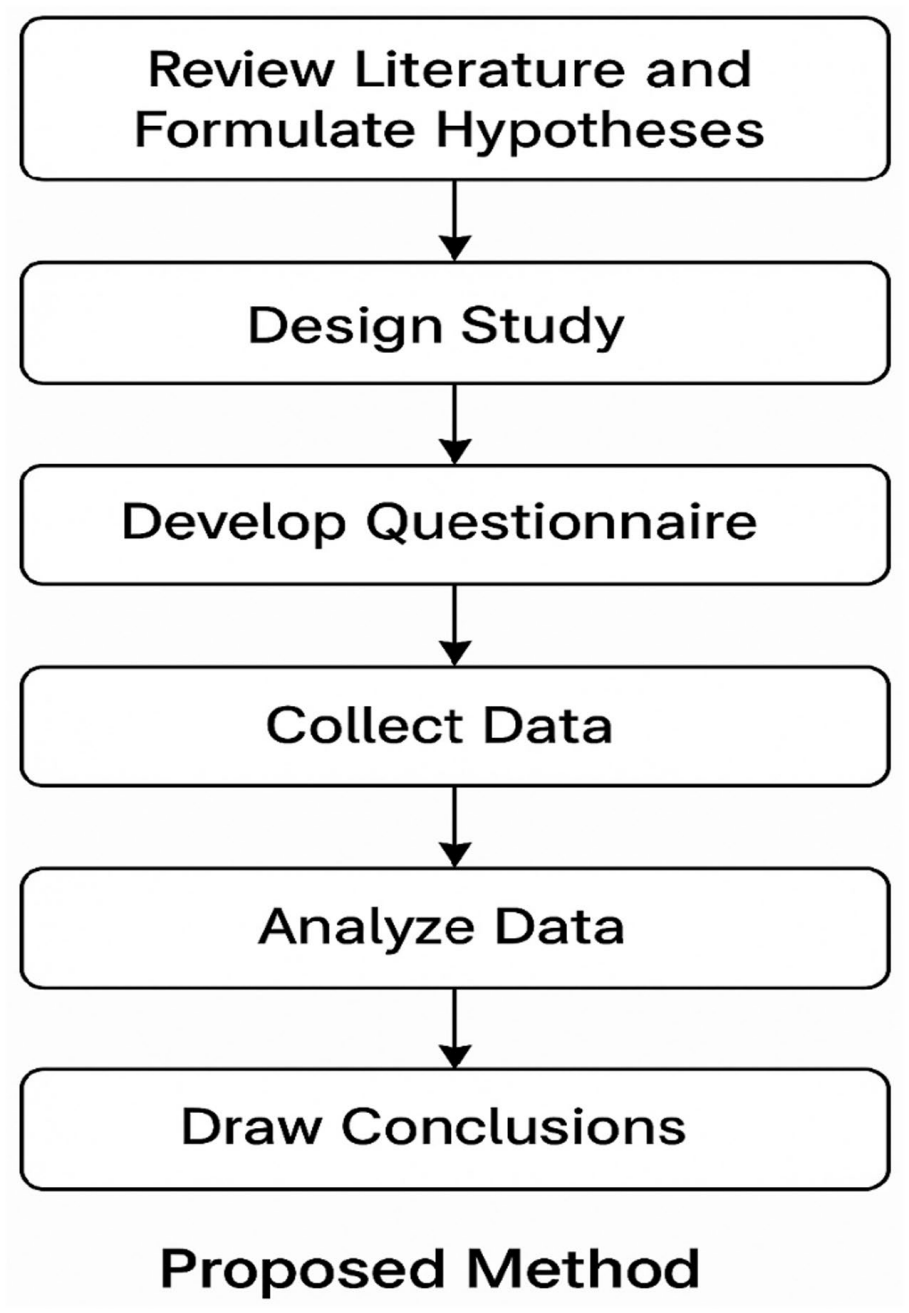
Workflow of the proposed research methodology.

To provide a simple overview of the research steps, a diagram (see Figure 1) has been included illustrating the workflow followed in this study. The process begins with designing the questionnaire, followed by distributing it to participants from Jordan and Saudi Arabia, then collecting and analyzing the responses, and finally presenting and discussing the results. This diagram helps clarify the sequence of methodological steps in a clear and straightforward manner.

### Study sample and its characteristics

The study sample consisted of (170) participants from employees working in the artificial intelligence sectors in both the Hashemite Kingdom of Jordan and the Kingdom of Saudi Arabia, divided into (85 participants from the Jordanian community, and 85 participants from the Saudi community) whose ages ranged between 35 and 58 years, with an average age of (45.2 years), Table 1 shows the demographic characteristics of the study sample.

**Table 1:** The demographic characteristics of the study sample.

M	Demographic variables		Jordanian society	Saudi society	Total
1	Sex	Male	56	51	107
		Female	29	34	63
		Total	85	85	170
2	Age	From 35 to 40 years old	26	23	49
		From 41 to 46 years old	30	21	51
		From 47 to 52 years old	16	20	36
		From 53 to 58 years old	13	21	34
		Total	85	85	170
3	Education Level	Doctorate “PhD”	10	5	15
		Master’s “MA”	17	11	28
		Bachelor’s “BA”	58	69	127
		Total	85	85	170
4	Use of Smart Devices	Partial use	58	67	125
		Full use	27	18	45
		Total	85	85	170

### Study tool

In collecting the field study data from its primary sources, the researchers relied on a questionnaire list that she prepared specifically for this purpose in light of the theoretical framework and previous studies, and in light of what the exploratory study revealed. This list includes a set of questions to measure the study variables represented by “the role of artificial intelligence in reducing crime and the challenges it faces, and the individual’s methods in confronting these challenges.” The following is a presentation of the steps for designing the questionnaire:

A. Defining the objective of the scale: The objective of the scale is to identify the role of artificial intelligence in reducing crime, the challenges it faces, and individual approaches to addressing these challenges.
B. Reviewing the theoretical frameworks and previous studies, both Arab and foreign, in the field of artificial intelligence, its role in reducing crime, the challenges it faces, and individual approaches to addressing these challenges.
C. The researchers prepared open-ended questions directed to a sample of randomly selected respondents from the Hashemite Kingdom of Jordan and the Kingdom of Saudi Arabia. These open-ended questions included their perspectives on: “From your perspective, what is the role of artificial intelligence in reducing crime?” and “From your perspective, what are the obstacles or challenges facing the application of artificial intelligence?
D. The respondents’ responses to the questions were carefully read, and the frequencies and percentages for all their answers were calculated. The researcher was then able to formulate a set of questions and phrases in a procedural, measurable format to express the role of artificial intelligence in reducing crime and the challenges facing its application.
E. The researchers took care in formulating the questionnaire’s initial phrases to be appropriate for the respondents to whom the scale would be applied. The phrases should be easy, clear, direct, and short, stimulating their mental and cognitive skills, not requiring a long time to respond, not carrying more than one meaning, measuring what they were designed to measure without ambiguity, and expressing different viewpoints.

The research type scale included three Likert scale as follows:

**Table:** 

No	Sometime	Yes
1	2	3

Relative importance, assigned due to:


$$\begin{array}{l} \text{Class Interval}=(\text{Maximum Class}-\text{Minimum Class})\\ ÷\text{Number of Levels}=(3-1)÷3=2÷3\approx 0.66 \end{array}$$

- The Low level from 1.00 to 1.66
- The Medium level from 1.67 to 2.33
- The High level from 2.34 to 3.00 F

F. The questionnaire, in its initial form, was presented to a group of university faculty members specializing in artificial intelligence, who provided their opinions and feedback on its scientific validity for application to the subjects.
G. The final form of the questionnaire consisted of four parts. The first part included information and data about the study sample members and their demographic and functional characteristics, including gender, age, educational level, and use of smart devices. The second part included statements indicating the role of artificial intelligence in reducing crime and it’s consisted of the statements from (1–12). The third part of the questionnaire addressed the obstacles or challenges facing the application of artificial intelligence and it’s consisted of the statements from (13–22), while the fourth part addressed the styles of coping artificial intelligence and its consisted of the statements from (23–33).

### Validity

The questionnaire was presented to specialists from the faculty members of the Jordanian universities to verify the validity of its paragraphs. The researcher benefited from the specialist’s observations by adopting the agreed upon observations at a rate of (80%), whether by deletion, addition or amendment until the study tool appeared in its final form, divided into three variables and each variable contains of a few statements. The researcher considered the opinions of the specialists and their adjustments as an indication of the truthfulness of the content of the study tool, the relevance and diversity of its paragraphs. After making the required adjustments, the balance between the contents of the study instrument in its paragraphs is achieved, thus the validity of the scale has verified.

### Reliability

To investigate from the reliability of the study tool, the study used two methods:

#### Cronbach’s alpha coefficient

To identify the consistency of each paragraph of the questionnaire with the dimension to which the paragraph belongs, the researcher used the correlation coefficients between each of the paragraphs in the questionnaire by using (Chronbach Alpha). Where the values of the Cronbach alpha of the study tool were generally higher than (0.70), which is an acceptable ratio (Hair et al., 2017) in research’s as in Table 2:

**Table 2:** Cronbach’s alpha for the study fields.

Variables	Statements	Cronbach alpha
The role of artificial intelligence in reducing crime	1–12	0.720
The obstacles or challenges facing the application of artificial intelligence	13–22	0.741
The styles of coping artificial intelligence	23–33	0.770
All statements	1–33	0.952

Source: prepared by the researcher.

#### Split—half

In this method, the scale is applied once to the examinees, then the test is divided into two equal parts and the correlation coefficient of the two parts is calculated. The half-split of the anxiety scale was calculated by using the following equations: “Spearman-Brown correlation coefficient, Gettman coefficient, Cronbach’s alpha coefficient.” Table 3 shows the stability coefficients of the individuals’ scores on the questionnaire of the current study using the half-split with the correlation coefficients of Spearman-Brown, Gettman, and Cronbach’s alpha.

**Table 3:** Stability coefficients of questionnaire scores using half-split and various correlation methods.

Artificial intelligence	Reliability statistics		
	Spearman-brown coefficient	Guttman split-half coefficient	Cronbach’s Alpha
Factor 1: The Role of Artificial Intelligence in Reducing Crime	0.83	0.79	0.720
Factor 2: Challenges Facing AI	0.79	0.76	0.741
Factor 3: Styles of Coping Artificial Intelligence	0.91	0.82	0.770

It is clear from Table 3 that: the values of the Spearman-Brown correlation coefficient, the Gutman correlation coefficient, and the Cronbach’s alpha correlation coefficient are considered high reliability values, which reassures the researcher to use this questionnaire in the current study.

#### Description of the scale study in its final form and the method of correcting it

The current study questionnaire in its final form consists of (33) statements to measure artificial intelligence. These statements are distributed over three dimensions. The first dimension aims to identify the role of artificial intelligence in reducing crime and is represented by statements from (1:12). The second dimension aims to identify the challenges facing the application of artificial intelligence and is represented by statements from (13:22). The third dimension aims to identify the individual’s methods in facing such challenges and is represented by statements from (23:33). All of these statements are responded to through a three-point scale consisting of “yes, sometimes, no.” These responses are corrected by giving three marks for the response (yes), two marks for the response (sometimes), and one mark for the response (no). This is for statements with a positive direction. As for statements with a negative direction, one mark is given for the response under the weight (yes), two marks for the response under the weight (sometimes), and three marks under the weight (no).

### Statistical methods used in processing study data

The researchers Relied on the Statistical Package for Social Sciences (SPSS) to conduct the statistical transactions and graphs used in the study. A number of statistical methods were used to analyze and process the data of the current study and test its hypotheses. These methods were represented in arithmetic means, standard deviations, and the T-test to calculate the significance of the differences between the means, and the correlation coefficient (Pearson, Spearman-Brown, and Cronbach’s Alpha).

### Presentation, interpretation and discussion of the study results

The First Hypothesis: Artificial Intelligence Plays a Significant Role in Reducing Crime.

The study used the arithmetic mean, standard deviation, item importance and importance level to show the level of the Role of Artificial Intelligence in Reducing Crime as shown in Table 4. And to verify this hypothesis, the researchers used One Sample T-test to show the Artificial Intelligence and its significant role in reducing crime, as following tables.

**Table 4:** Arithmetic mean, SD, item importance and importance level of the role of AI in reducing crime in descending order.

No	Statements	Mean	Std. deviation	Item importance	Importance level
1	Artificial intelligence techniques can identify the suspected criminal.	2.94	0.27	1	High
2	Artificial intelligence techniques can accurately map the crime scene.	2.89	0.35	2	High
11	Artificial intelligence reduces the time required to detect crimes.	2.86	0.42	3	High
4	Artificial intelligence techniques can prove whether or not an accused person is capable of committing the behavior that constitutes a crime.	2.85	0.39	4	High
7	Artificial intelligence technologies can analyze smartphone data and monitor criminal calls.	2.81	0.43	5	High
3	Artificial intelligence technologies can identify the real perpetrator of a crime and gather information about criminals.	2.79	0.41	6	High
12	Artificial intelligence contributes to maintaining security.	2.72	0.49	7	High
10	Artificial intelligence technologies can prevent crimes before they happen and deter criminal activity.	2.70	0.48	8	High
5	Artificial intelligence technologies can accurately recognize facial data of criminals.	2.66	0.49	9	High
8	Artificial intelligence technologies can track various crimes quickly and accurately.	2.62	0.53	10	High
9	Artificial intelligence technologies can detect danger areas.	2.62	0.62	11	High
6	Artificial intelligence technologies can accurately analyze audio recordings of perpetrators.	2.59	0.58	12	High
Total		2.75	0.21		High

It was found through the responses of the participants in Table 4 showed that the means of this dimension (Role of AI in Reducing Crime), ranged between (2.94–2.59), where the whole dimension earned a total mean of (2.75), which is a level of High. Statement (1) (Artificial intelligence techniques can identify the suspected criminal) ranked first with mean of (2.94), and with standard deviation of (0.27), which is a level of high, and statement (2) (Artificial intelligence techniques can accurately map the crime scene) ranked second with mean of (2.89), and standard deviation of (0.35), which is of a high level also.

Statement (6) (Artificial intelligence technologies can accurately analyze audio recordings of perpetrators) ranked last with mean of (2.59), and a standard deviation of (0.58), which is of a high level.

One Sample T-Test was used to confirm that artificial intelligence plays a role in reducing the level of crime.

Table 5 showed that the mean value for the Role of Artificial intelligence in reducing crime was (2.75), which is a higher value than the default scale (1.5), and the standard deviation value was (0.21). (t) calculated value was of (79.327), which is a higher value than the tabulated (t) value, and the results showed the presence of statistically significant differences at (0.05) between the mean value of the scale and default mean value, which indicates the acceptance of the study hypothesis, which states: Artificial Intelligence Plays a Significant Role in Reducing Crime.

**Table 5:** One sample T-test to show the role of artificial intelligence in reducing crime.

Mean	Standard deviation	T calculated	T tabulated	Df	Sig.
2.75	0.21	79.327	1.96	169	0.00^*^

* Significant at level of (0.05) (t value = 1.5).

The Second Hypothesis: There are Challenges Facing Artificial intelligence in reducing crime.

The study used the arithmetic mean, standard deviation, item importance and importance level to show the level of the challenges facing artificial intelligence in reducing crime as shown in Table 6. And to verify this hypothesis, the researchers used One Sample T-test to show the challenges facing artificial intelligence in reducing crime, as following tables.

**Table 6:** Arithmetic mean, SD, item importance and importance level of the challenges facing artificial intelligence in reducing crime.

No	Statements	Mean	Std. deviation	Item importance	Importance level
18	Research centers specializing in artificial intelligence are not available in all sectors.	2.79	0.43	1	High
14	I believe that AI systems violate individuals’ privacy.	2.66	0.51	2	High
20	I believe that the training provided in digital skills and smart systems is insufficient to deal with artificial intelligence technologies.	2.66	0.51	3	High
22	I believe there are not enough highly trained professionals to deal with AI technologies.	2.64	0.53	4	High
15	I believe that AI systems are outside of legal accountability and are not subject to any law.	2.62	0.60	5	High
17	I believe that the means of sharing and exchanging information with different parties may be insecure.	2.62	0.59	6	High
13	I believe that intelligent artificial intelligence systems will replace the human element.	2.51	0.58	7	High
19	I find it very difficult to deal efficiently with artificial intelligence technologies.	2.48	0.65	8	High
21	The culture of collaboration with colleagues on how to efficiently handle AI technologies is largely lacking.	2.34	0.76	9	High
16	Reliance on artificial intelligence will reduce job opportunities for humans in the future, and many will lose their jobs.	1.96	0.77	10	Medium
Total		2.53	0.26		High

It was found through the responses of the participants in Table 6 showed that the means of this dimension (challenges facing artificial intelligence in reducing crime), ranged between (2.79–1.96), where the whole dimension earned a total mean of (2.53), which is a level of High. Statement (18) (Research centers specializing in artificial intelligence are not available in all sectors.) ranked first with mean of (2.79), and with standard deviation of (0.43), which is a level of high, and statement (14) (I believe that AI systems violate individuals’ privacy) ranked second with mean of (2.66), and standard deviation of (0.51), which is of a high level also.

Statement (16) (Reliance on artificial intelligence will reduce job opportunities for humans in the future, and many will lose their jobs) ranked last with mean of (1.96), and a standard deviation of (0.77), which is of a medium level.

One Sample T-Test was used to confirm that challenges facing artificial intelligence in reducing crime.

Table 7 showed that the mean value for the challenges facing Artificial intelligence in reducing crime was (2.53), which is a higher value than the default scale (1.5), and the standard deviation value was (0.26). (t) calculated value was of (50.919), which is a higher value than the tabulated (t) value, and the results showed the presence of statistically significant differences at (0.05) between the mean value of the scale and default mean value, which indicates the acceptance of the study hypothesis, which states: There are Challenges Facing Artificial intelligence in reducing crime.

**Table 7:** One sample T-test to show the challenges facing artificial intelligence in reducing crime.

Mean	Standard Deviation	T calculated	T tabulated	Df	Sig.
2.53	0.26	50.919	1.96	169	0.00^*^

* Significant at level of (0.05) (t value = 1.5).

The Third Hypothesis: There are Styles Coping Artificial intelligence in reducing crime.

The study used the arithmetic mean, standard deviation, item importance and importance level to show the level of the style coping artificial intelligence in reducing crime as shown in Table 8. And to verify this hypothesis, the researchers used One Sample T-test to show the challenges facing artificial intelligence in reducing crime, as following tables.

**Table 8:** Arithmetic mean, SD, item importance and importance level of the style coping artificial intelligence in reducing crime.

No	Statements	Mean	Std. deviation	Item importance	Importance level
32	I have the ability to find multiple solutions to address problems related to artificial intelligence technologies in my work	2.86	0.42	1	High
33	I face artificial intelligence challenges with resilience	2.76	0.44	2	High
25	I read specialized books on artificial intelligence	2.75	0.49	3	High
28	I make sure to receive training related to artificial intelligence	2.62	0.58	4	High
29	I confront problems related to artificial intelligence technologies regardless of the challenges	2.61	0.59	5	High
27	I consider artificial intelligence technologies as a challenge to my abilities	2.55	0.63	6	High
23	I seek the necessary information to learn how to work with artificial intelligence technologies.	2.50	0.64	7	High
24	I constantly engage in discussions with specialists in artificial intelligence technologies.	2.48	0.65	8	High
26	I talk with colleagues who have more experience in working with artificial intelligence technologies	2.41	0.63	9	High
31	I exert my utmost effort to develop my skills and capabilities in dealing with artificial intelligence technologies	2.39	0.73	10	High
30	I need a considerable amount of time to overcome the challenges of artificial intelligence	2.31	0.78	11	High
Total		2.57	0.27		High

It was found through the responses of the participants in Table 8 showed that the means of this dimension (style coping artificial intelligence in reducing crime), ranged between (2.86–2.31), where the whole dimension earned a total mean of (2.57), which is a level of High. Statement (32) (I have the ability to find multiple solutions to address problems related to artificial intelligence technologies in my work) ranked first with mean of (2.86), and with standard deviation of (0.42), which is a level of high, and statement (33) (I face artificial intelligence challenges with resilience) ranked second with mean of (2.76), and standard deviation of (0.44), which is of a high level also.

Statement (30) (I need a considerable amount of time to overcome the challenges of artificial intelligence) ranked last with mean of (2.31), and a standard deviation of (0.78), which is of a high level.

One Sample T-Test was used to confirm that challenges facing artificial intelligence in reducing crime.

Table 9 showed that the mean value for the style coping Artificial intelligence in reducing crime was (2.57), which is a higher value than the default scale (1.5), and the standard deviation value was (0.27). (t) calculated value was of (51.145), which is a higher value than the tabulated (t) value, and the results showed the presence of statistically significant differences at (0.05) between the mean value of the scale and default mean value, which indicates the acceptance of the study hypothesis, which states: There are Styles Coping Artificial intelligence in reducing crime.

**Table 9:** One sample T-test to show the style coping artificial intelligence in reducing crime.

Mean	Standard deviation	T calculated	T tabulated	Df	Sig.
2.57	0.27	51.145	1.96	169	0.00^*^

* Significant at level of (0.05) (t value = 1.5).

The Fourth Hypothesis: There are differences between individuals in Jordanian society and Saudi society in overcoming the challenges of artificial intelligence.

To verify the validity of the hypothesis, the researchers calculated the t-test value for the responses of the individuals participating in the study sample to statements indicating methods for facing the challenges of artificial intelligence. Table 10 shows the significance of the differences between the average scores of Jordanian and Saudi society in overcoming the challenges of artificial intelligence.

**Table 10:** Significance of differences in overcoming AI challenges between Jordanian and Saudi societies.

Variable	Society	N	Mean	Standard deviation	T	Sig
Challenges of artificial intelligence	Jordanian	85	2.50	0.27	**−1.549**	0.123
	Saudi	85	2.56	0.26		

Bold values indicate the calculated t-value and its corresponding significance level (Sig) used to test the differences between the two groups.

It is clear from Table 10 that: There are no statistically significant differences between the average scores of the Jordanian society and the Saudi society in overcoming the challenges of artificial intelligence, (t) value calculated = (−1.549) and its not significant at level of (0.05) and the variance between means values if its fount, but its not significant at level of (0.05).

**Figure 2:**
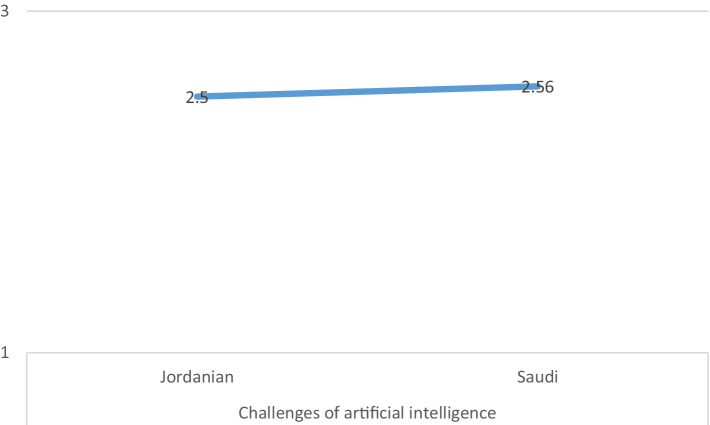
Differences in overcoming ai challenges between Jordanian and Saudi societies.

Figure 2 shows the differences between the average scores of Jordanian society and Saudi society in overcoming the challenges of artificial intelligence.

## Discussion of results

Looking at Table 4 it is clear that the estimates and responses of the participating individuals from the study sample regarding the role of artificial intelligence in reducing crime were high. At the forefront of these roles, according to their relative weights, was that artificial intelligence contributes to maintaining security, and that artificial intelligence technologies can identify the identity of the suspected criminal, followed by discovering the danger spots, then accurately drawing the crime scene, identifying the real perpetrator of the crime, collecting information about criminals, accurately analyzing the audio recordings of criminals, as well as analyzing smartphone data and monitoring criminal calls, preventing crimes before they occur and deterring criminal activity, and that artificial intelligence reduces the time required to detect crimes, then accurately recognizes the facial data of the criminal. Artificial intelligence technologies can also prove the extent to which the accused is capable of committing the behavior that constitutes the crime or not, in addition to its ability to track various crimes quickly and accurately.

Looking at Table 6, it is clear that the study sample’s assessments and responses regarding the challenges of artificial intelligence were consistent. The most prominent of these, according to the arithmetic averages, were: specialized AI research centers are not available in all sectors; AI systems violate individuals’ privacy; the training provided in digital skills and smart systems is insufficient to handle AI technologies; there are insufficient highly trained professionals to handle AI technologies; AI systems are not subject to legal accountability and are not subject to any law; the means of sharing and exchanging information with various parties may be unsafe; intelligent AI systems will replace humans; and the challenges of dealing efficiently with AI technologies are extremely difficult.

The results of Table 8 showed that the approach to dealing with artificial intelligence in reducing crime is at a high level. It was found that the study sample members have the ability to find multiple solutions to address problems related to artificial intelligence technologies in their work. They also face the challenges of artificial intelligence with flexibility, read specialized books on artificial intelligence, and are keen to receive training related to it. They also face problems related to artificial intelligence technologies regardless of the challenges. They express that artificial intelligence technologies are a challenge to their abilities and seek to obtain the necessary information to learn how to work with artificial intelligence technologies.

Researchers find no statistically significant differences between Jordanian and Saudi societies in overcoming the challenges of artificial intelligence, which could reduce crime rates. This may be due to the similarity of security and legislative policies in both countries, and the existence of national strategies that support digital transformation and the use of AI technologies in security fields. This convergence is also attributed to the availability of a relatively similar digital infrastructure and a clear government orientation toward integrating AI into surveillance systems, forensic analysis, and crime prediction.

Recent regional studies have shown that both Saudi Arabia and Jordan have adopted projects that rely on artificial intelligence technologies to analyze criminal data and monitor suspicious behavior online. These projects include the use of machine learning algorithms to track cybercrimes and monitor social media to detect security threats.

For example, Saudi Arabia launched the Data and Artificial Intelligence Authority (SDAIA), which is working in collaboration with the Ministry of Interior on smart projects to analyze crimes before they occur, using massive data from smart cameras and traffic systems. In contrast, Jordan has begun implementing programs based on security data analysis to allocate security resources more effectively, especially in densely populated urban areas.

Despite some challenges, such as a lack of AI-specific skills, privacy considerations, and ethical concerns, both societies are moving at a similar pace in employing this technology to reduce crime, which explains why there are no significant differences between them in this context.

## Conclusion

Based on the study’s results and the validation of its hypotheses, the researchers conclude that artificial intelligence plays a vital role in reducing crime despite several obstacles. AI enables timely monitoring and detection of criminal activities and threats, predicts and prevents crimes, identifies suspects accurately, analyzes crime scenes, and supports justice processes, thereby enhancing community safety.

The study also reveals significant differences between Jordanian and Saudi professionals in their ability to cope with AI-related challenges, favoring Jordanians. This disparity likely stems from Jordan’s comprehensive digital policies, ethical AI frameworks, investment in training, and strategic initiatives fostering AI literacy and infrastructure.

These findings underscore the urgent need to enhance training programs, develop robust legal oversight, and promote collaborative cultures to maximize AI’s potential in crime reduction. Future research should focus on context-specific strategies and evaluate long-term impacts to guide effective AI integration in public security.

## Recommendations and proposed research

Drawing from the findings of this study, the researchers put forward the following recommendations:

1. Promote further research and studies on artificial intelligence, focusing on its growing significance across all sectors and its expanding impact on different areas of life. Increase the number of specialized research centers dedicated to these technologies. Encourage widespread adoption of AI technologies within the Kingdom by addressing and removing any potential barriers. Additionally, establish an annual conference that brings together local, regional, and international researchers and stakeholders to share the latest advancements in AI.
2. Develop a strategic plan for the Kingdom aimed at training and improving the skills of employees in the effective use of artificial intelligence technologies. This plan should include secure and dependable methods for information sharing and exchange among various parties. It should also provide shared repositories and reliable, continuously updated data sources, make use of open APIs and cloud platforms, and support the localization of digital products. Moreover, it should ensure the availability of a sufficient number of well-trained specialists capable of managing these technologies across all business sectors.
3. Expand awareness programs targeting all members of society to highlight the role of smart systems and AI technologies in crime reduction. Emphasize that these systems operate within a regulated legal framework, which is regularly reviewed to address potential risks and ensure accountability.

## Data Availability

The original contributions presented in the study are included in the article/supplementary material, further inquiries can be directed to the corresponding author.
