# Correction: Artificial intelligence as a tool to enhance social interventions in reducing crime

**DOI:** 10.3389/frai.2025.1718188

**Published:** 2025-11-06

**Authors:** Haya H. Tarawneh, Niveen Z. Halalsheh, Bahjat Abu Sulaiman, Huda A. Alhajjaj, Nesreen N. Atieh

**Affiliations:** 1 The University of Jordan, Aljubeiha, Jordan; 2 Department of Social Work, Faculty of Arts, The University of Jordan, Amman, Jordan; 3 Department of Journalism, Media and Digital Communication, School of Arts, The University of Jordan, Amman, Jordan; 4 Educational and Psychological Counseling, Saudi Arabia, Saudi Arabia

**Keywords:** artificial intelligence, social interventions, crime, enhance social, tool

Author Niveen Halalsheh was erroneously assigned to affiliation Department of Social Work, Faculty of Arts, The University of Jordan, Amman, Jordan. The correct affiliation is Department of Journalism, Media and Digital Communication, School of Arts, The University of Jordan, Amman, Jordan. This affiliation has now been replaced for author Niveen Halalsheh.

Author Niveen Z. Halalsheh was erroneously spelled as Niveen Halalsheh.

Author Huda A. Alhajjaj were erroneously spelled as Huda Alhajjaj.

The original version of this article has been updated.

